# Catarrhal Fever

**Published:** 1879-03-20

**Authors:** 


### *CATARRHAL FEVER.*

The extreme cold weather which prevailed early in the month of January last produced in this section and through the Southern States, a singular and very obstinate form of catarrhal fever. In Atlanta the thermometer indicated at one time only 5° F. above zero, and for several days stood at 8° and 10°. When it is remembered that it is a rare thing in this section to see a temperature of 20°, and that only for a day or two, and that the mercury had not since 1835 reached so low a point, and that so many days of extreme cold had never before been experienced by the larger portion of our citizens now living, it is not surprising that it was followed by bronchial and lung troubles. The form of disease which resulted was that of a deep bronchial irritation and in many cases inflammation, attended with severe and prolonged paroxyisms of coughing, particularly at night. In many cases there were daily exascerbations of fever accompanied with some neuralgic pains in the chest, head and other parts of the body. The ordinary cough mixtures made little or no impression on the disease. Some relief was obtained from quinine and dovers powder, and aconite and gelseminum during the fever, and revulsives to the chest. In most cases the cough continued with varied degrees of severity from six to eight weeks.

